# Obesity Indices for Predicting Functional Fitness in Children and Adolescents With Obesity

**DOI:** 10.3389/fped.2021.789290

**Published:** 2021-12-15

**Authors:** Kanokporn Udomittipong, Teerapat Thabungkan, Akarin Nimmannit, Prakarn Tovichien, Pawinee Charoensitisup, Khunphon Mahoran

**Affiliations:** ^1^Division of Pulmonology, Department of Pediatrics, Faculty of Medicine Siriraj Hospital, Mahidol University, Bangkok, Thailand; ^2^Division of Clinical Epidemiology, Office for Research and Development, Faculty of Medicine Siriraj Hospital, Mahidol University, Bangkok, Thailand

**Keywords:** obesity indices, functional fitness, children, adolescents, 6-MWT, spirometry, respiratory muscle strength, obesity

## Abstract

**Objectives:** We aimed to determine the obesity indices that affect 6-min walk test (6-MWT) distance in children and adolescents with obesity and to compare the 6-MWT distance of obese subjects with that of normal-weight subjects.

**Methods:** Obese children and adolescents aged 8–15 years and normal-weight age- and gender-matched controls were enrolled. All participants performed the 6-MWT; respiratory muscle strength (RMS), including maximal inspiratory pressure and maximal expiratory pressure; and spirometry. Data between groups were compared. In the obesity group, correlation between obesity indices and pulmonary function testing (6-MWT, RMS, and spirometry) was analyzed.

**Results:** The study included 37 obese and 31 normal-weight participants. The following parameters were all significantly lower in the obesity group than in the normal-weight group: 6-MWT distance (472.1 ± 66.2 vs. 513.7 ± 72.9 m; *p* = 0.02), forced expiratory volume in one second/forced vital capacity (FEV_1_/FVC) (85.3 ± 6.7 vs. 90.8 ± 4.5%; *p* < 0.001), forced expiratory flow rate within 25–75% of vital capacity (FEF_25−75%_) (89.8 ± 23.1 vs. 100.4 ± 17.3 %predicted; *p* = 0.04), and peak expiratory flow (PEF) (81.2 ± 15 vs. 92.5 ± 19.6 %predicted; *p* = 0.01). The obesity indices that significantly correlated with 6-MWT distance in obese children and adolescents were waist circumference-to-height ratio (WC/Ht) (*r* = −0.51; *p* = 0.001), waist circumference (*r* = −0.39; *p* = 0.002), body mass index (BMI) (*r* = −0.36; *p* = 0.03), and chest circumference (*r* = −0.35; *p* = 0.04). WC/Ht was the only independent predictor of 6-MWT distance by multiple linear regression.

**Conclusions:** Children and adolescents with obesity had a significantly shorter 6-MWT distance compared with normal-weight subjects. WC/Ht was the only independent predictor of 6-MWT distance in the obesity group.

## Introduction

Obesity is now a major global public health problem among both children and adults, and its prevalence continues to increase (1–3). Some obese individuals experience shortness of breath during exercise or physical activities, which is caused by impaired lung function. Most previous studies of pulmonary function testing (PFT) in children and adolescents with obesity used spirometry or lung volume testing, neither of which is measured during physical activity. The 6-minute walk test (6-MWT) is a PFT that measures how many meters a person can walk in 6 min. This test is simple and inexpensive, and it is both a cause of fatigue and a method for helping to measure it in obese individuals. It is also helpful for assessing functional capacity and an individual's ability to perform activities of daily living (4, 5). Therefore, the 6-MWT could be a good measurement tool for assessing and following up functional capacity in obese individuals who are easily tired during exercise. Most studies of the 6-MWT were conducted in adults, with only a few conducted in obese children and adolescents (6–11).

Studies specific to obesity indices that influence 6-MWT distance in children and adolescents with obesity are scarce. In addition, most studies (6–9, 11) included body weight (BW), body mass index (BMI), BMI *z*-score, chest circumference (CC), neck circumference (NC), hip circumference (HC), and/or waist circumference (WC) as obesity index variables, but they rarely included waist circumference-to-height ratio (WC/Ht), which is an abdominal obesity index. To our knowledge, only a study by Axley et al. (10) included WC/Ht in addition to other obesity indices, and they reported it to be the best determinant of 6-MWT distance.

A recent systematic review (12) and previous studies in adults (13–16) reported abdominal obesity indices to be better predictors of impaired lung function than BMI, which reflects overall obesity, not specific body fat distribution. In addition to the WC/Ht being previously reported to be a significant predictor of pulmonary function, it was also reported to be a significant predictor of cardiovascular and metabolic risk in children, adolescents, and adults with obesity (17–19).

The primary aim of this study was to determine the obesity indices that influence 6-MWT distance in obese children and adolescents by including WC/Ht as a variable. The secondary objective was to compare 6-MWT distance between children and adolescents with obesity and normal-weight children and adolescents. Lastly, in contrast to most previously published studies, we included 6-MWT distance, respiratory muscle strength (RMS), and spirometry as PFTs, and we set forth to determine the obesity indices that affect these commonly used PFTs. Those results were then compared between the obese and normal-weight groups.

## Materials and Methods

### Study Population

This cross-sectional study recruited children and adolescents aged 8–15 years who were diagnosed as obese and a control group of normal-weight age- and gender-matched children and adolescents. Obesity was defined as a BMI *z*-score ≥2, and normal weight was defined as a BMI *z*-score between −1 and 1 according to the World Health Organization (WHO) reference criteria (20). Exclusion criteria included a history of pulmonary, cardiac, or neuromuscular diseases; history of smoking or environmental tobacco smoke; respiratory infection during the past 4 weeks; and/or inability to perform the proposed tests. Participant age, gender, height, and obesity indices, including BW, BMI, BMI z-score, CC, WC, and WC/Ht, were collected. The PFTs (6-MWT, RMS, and spirometry) were performed by the same trained technician.

This study was conducted at the Division of Pulmonology of the Department of Pediatrics, Faculty of Medicine Siriraj Hospital, Mahidol University, Bangkok, Thailand, during October 2020 to May 2021. The study protocol was approved by our center's institutional review board (IRB) (approval no. Si 848/2020). Informed consent or assent (when applicable) was obtained from participants and/or their legal guardians before enrollment in the study.

### Anthropometric Evaluation

Body weight and height were determined using standard scale (TANITA Corporation, Tokyo, Japan). BMI was calculated as body weight (kg) divided by the square of height (m^2^). BMI was also expressed as *z*-score (BMI z-score) with adjustments for age and gender according to the WHO growth reference (21, 22). WC was measured between the inferior margin of the last rib and the iliac crest (23), and CC was measured at nipple level with a measuring band. The WC/Ht was calculated as WC (cm)/height (cm).

### 6-MWT

The 6-MWT was performed according to the guidelines of the American Thoracic Society (24). Participants were instructed to perform the 6-MWT by walking as fast as they could without running for 6 min on a flat and straight surface. The walking course was 30 m in length, with cones placed at each end of the course. Subjects were allowed to stop and rest during the test if they needed to, but they were instructed to continue walking as soon as they were capable of doing so. Participants were given words of encouragements during the test, and the time remaining was announced at different time points during the test. The distance walked was measured after 6 min of walking. Participant heart rate, respiratory rate, blood pressure, and peripheral oxygen saturation (SpO2%) were measured before the test after a resting period in sitting position for at least 10 min and immediately after the test. The test was immediately stopped if any participant complained of chest pain, shortness of breath, or heart palpitations.

### RMS

RMS evaluation was performed by measuring the maximal inspiratory and expiratory pressure (MIP and MEP, respectively) using a Vyntus™ BODY instrument (Vyaire Medical, Mettawa, IL, USA) according to American Thoracic Society and European Respiratory Society (ATS/ERS) recommendations (25).

MIP was measured during maximum inspiration to the level of total lung capacity following maximum expiration, and MEP was measured during forced expiration to the level of residual volume, following maximum inspiration. A maximum of 9 maneuvers for each MIP and MEP assessment was performed (26). At least 3 acceptable measurements (without leakage and lasting for at least 1 s) were obtained, and at least 2 were reproducible (a difference between them of 10% of the highest value). The measurement with the highest value was recorded. However, if the highest value was obtained on the last maneuver, the test was repeated until a value with a difference of 10% was obtained (25). The MIP and MEP values were expressed in absolute and %predicted values according to the reference equations by Verma et al. (27).

### Spirometry

Spirometry was performed using a Vyntus™ BODY instrument (Vyaire Medical) according to ATS/ERS recommendations (28).

Spirometry data were collected, including forced vital capacity (FVC), forced expiratory volume in 1 s (FEV_1_), FEV_1_/FVC ratio, forced expiratory flow rate within 25–75% of vital capacity (FEF_25−75%_), and peak expiratory flow (PEF). All parameters, excluding FEV_1_/FVC ratio, were expressed as a percentage of predicted value based on reference values from multi-ethnic global lung function (2012) equations (29).

### Statistical Analysis

The data are presented as mean plus/minus standard deviation or number and percentage depending on whether the data are continuous or categorical. Collected data were compared using Student's *t*-test for continuous data and using chi-square test or Fisher's exact test for categorical data. The correlations between PFTs (6-MWT, RMS, and spirometry) and obesity indices (weight, BMI, BMI *z*-score, CC, WC, and WC/Ht) in the obese group were analyzed using Pearson's correlation test, and the results are presented as Pearson's correlation coefficients (*r*). Multiple linear regression analysis was then employed to determine the obesity indices that influence 6-MWT distance, RMS, and spirometry. SPSS Statistics for Windows, version 18.0 (SPSS, Inc., Chicago, IL, USA), was used to analyze the data, and a *p*-value <0.05 was considered statistically significant.

## Results

The study enrolled 68 participants. Of those, 37 children and adolescents were obese, and the other 31 were of normal weight. There was no significant difference in age or gender between the two groups. Obesity indices, including BW (80.2 ± 25.9 vs. 39.8 ± 8.4 kg; *p* < 0.001), BMI (33.7 ± 6.5 vs. 17.92 ± 2.03 kg/m^2^; *p* < 0.001), BMI *z*-score (3.75 ± 0.93 vs. 0.25 ± 0.73; *p* < 0.001), CC (99.3 ± 14.57 vs. 71.5 ± 6.73 cm; *p* < 0.001), WC (104.9 ± 13.35 vs. 70 ± 6.95 cm; *p* < 0.001), and WC/Ht (0.68 ± 0.06 vs. 0.43 ± 0.15; *p* < 0.001), were significantly greater in the obesity group than in the normal-weight group. Obese children and adolescents were also significantly taller than normal-weight children (Table 1).

**Table 1:** Demographic data and obesity indices compared between the normal-weight and obesity groups.

**Data**	**Normal-weight group (*n* = 31)**	**Obesity group (*n* = 37)**	***p*-value**
Age (years), mean ± SD	11.26 ± 1.89	11.62 ± 2.06	0.46
Male gender, *n* (%)	16 (51.6%)	22 (59.5%)	0.62
Height (m), mean ± SD	1.47 ± 0.11	1.53 ± 0.11	***0.048***
Body weight (kg), mean ± SD	39.8 ± 8.4	80.2 ± 25.9	*** <0.001***
BMI (kg/m^2^), mean ± SD	17.92 ± 2.03	33.7 ± 6.5	*** <0.001***
BMI z-score, mean ± SD	0.25 ± 0.73	3.75 ± 0.93	*** <0.001***
Chest circumference (cm), mean ± SD	71.5 ± 6.73	99.3 ± 14.57	*** <0.001***
Waist circumference (cm), mean ± SD	70.0 ± 6.95	104.9 ± 13.35	*** <0.001***
WC/Ht, mean ± SD	0.43 ± 0.15	0.68 ± 0.06	*** <0.001***

*A p-value <0.05 indicates statistical significance. SD, standard deviation; BMI, body mass index, WC/Ht, waist circumference-to-height ratio. The bold italic means a p-value < 0.05*.

A comparison of 6-MWT distance, spirometry, and RMS between the obese and normal-weight groups revealed that the obesity group had a significantly shorter 6-MWT distance (472.1 ± 66.2 vs. 513.7 ± 72.9 m; *p* = 0.02), lower FEV_1_/FVC (85.3 ± 6.7 vs. 90.8 ± 4.5%; *p* < 0.001), lower FEF_25−75%_ (89.8 ± 23.1 vs. 100.4 ± 17.3 %predicted; *p* = 0.04), and lower PEF (81.2 ± 15.0 vs. 92.5 ± 19.6 %predicted; *p* = 0.01) compared to the normal-weight group (Table 2).

**Table 2:** 6-MWT distance, RMS, and spirometry compared between the normal-weight and obesity groups.

**Pulmonary function variables**	**Normal-weight group (*n* = 31)**	**Obesity group (*n* = 37)**	***p*-value**
6-MWT distance (m), mean ± SD	513.7 ± 72.9	472.1 ± 66.2	***0.02***
MIP (cmH_2_O), mean ± SD	88.3 ± 23.5	94.3 ± 25.1	0.31
MIP (%predicted), mean ± SD	101.6 ± 27.13	107.4 ± 31.9	0.43
MEP (cmH_2_O), mean ± SD	80.3 ± 21.5	77.4 ± 20.5	0.57
MEP (%predicted), mean ± SD	85.5 ± 20.3	82.3 ± 24.7	0.56
FVC (%predicted), mean ± SD	102.8 ± 12.0	108.9 ± 15.16	0.07
FEV_1_ (%predicted), mean ± SD	103.9 ± 11.0	103.4 ± 15.25	0.62
FEV_1_/FVC (%), mean ± SD	90.8 ± 4.5	85.3 ± 6.7	*** <0.001***
FEF_25−75%_ (%predicted), mean ± SD	100.4 ± 17.3	89.8 ± 23.1	***0.04***
PEF (%predicted), mean ± SD	92.5 ± 19.6	81.2 ± 15.0	***0.01***

*A p-value <0.05 indicates statistical significance. 6-MWT distance, 6-minute walk test distance; SD, standard deviation; MIP, maximal inspiratory pressure; MEP, maximal expiratory pressure; FVC, forced vital capacity; FEV_1_, forced expiratory volume in 1 s; FEF_25−75%_, forced expiratory flow rate within 25–75% of vital capacity; PEF, peak expiratory flow. The bold italic means a p-value < 0.05*.

Regarding association between lung function (6-MWT distance, RMS, and spirometry) and obesity indices in the obesity group, the 6-MWT distance was found to be negatively correlated with BMI (*r* = −0.36, *p* = 0.03), BMI *z*-score (*r* = −0.32, *p* = 0.06), CC (*r* = −0.35, *p* = 0.04), WC (*r* = −0.39, *p* = 0.002), and WC/Ht (*r* = −0.51, *p* = 0.001). However, the 6-MWT distance had no relationship with height or body weight. The %predicted MEP was negatively correlated with BMI, CC, and WC/Ht. No spirometric variables or other RMS-related variables were significantly correlated with any obesity indices (Tables 3, 4).

**Table 3:** Correlation between obesity indices (weight, BMI, and BMI *z*-score) and 6-MWT distance, RMS, and spirometry in the obesity group.

**Pulmonary function variables**	**Height (m)**		**BW (kg)**		**BMI (kg/m** ^**2**^ **)**		**BMI** ***z*****-score**	
	***r***	***p-*value**	***r***	***p-*value**	***r***	***p-*value**	***r***	***p-*value**
6-MWT distance (m)	−0.06	0.69	−0.25	0.14	−0.36	0.03^**^	−0.32	0.06^*^
MIP (cmH_2_O)	−0.002	0.98	−0.03	0.86	−0.04	0.79	0.11	0.50
MIP (%predicted)	−0.18	0.28	−0.24	0.15	−0.22	0.29	0.004	0.98
MEP (cmH_2_O)	0.13	0.43	−0.02	0.99	−0.12	0.49	−0.002	0.99
MEP (%predicted)	−0.08	0.62	−0.26	0.12	−0.34	0.04^**^	−0.17	0.31
FVC (%predicted)	−0.12	0.46	0.01	0.94	0.13	0.44	0.10	0.53
FEV_1_ (%predicted)	−0.18	0.26	−0.06	0.70	0.03	0.87	0.12	0.47
FEV_1_/FVC	−0.16	0.32	−0.14	0.41	−0.13	0.43	−0.03	0.84
FEF_25−75%_ (%predicted)	−0.15	0.38	−0.15	0.38	−0.16	0.34	−0.11	0.54
PEF (%predicted)	−0.23	0.16	−0.11	0.54	−0.04	0.82	−0.15	0.93

*A p-value < 0.05 indicates statistical significance (*

* *p = 0.05–0.1*,

** *p < 0.05). BMI, body mass index; r, correlation coefficient; 6-MWT distance, 6-minute walk test distance; SD, standard deviation; MIP, maximal inspiratory pressure; MEP, maximal expiratory pressure; FVC, forced vital capacity; FEV1, forced expiratory volume in 1 s; FEF_25−75%_, forced expiratory flow rate within 25–75% of vital capacity; PEF, peak expiratory flow. The bold italic means a p-value < 0.1*.

**Table 4:** Correlation between obesity indices (CC, WC, and WC/Ht) and 6-MWT distance, RMS, and spirometry in the obesity group.

**Pulmonary function variables**	**CC (cm)**		**WC (cm)**		**WC/Ht**	
	***r***	***p-*value**	***r***	***p-*value**	***r***	***p-*value**
6-MWT distance (m)	−0.35	0.04^**^	−0.39	0.002^**^	−0.51	0.001^**^
MIP (cmH_2_O)	0.26	0.88	−0.06	0.72	−0.08	0.64
MIP (%predicted)	−0.22	0.20	−0.24	0.15	−0.18	0.27
MEP (cmH_2_O)	−0.08	0.96	−0.02	0.89	−0.15	0.37
MEP (%predicted)	−0.30	0.07^*^	−0.26	0.13	−0.29	0.07^*^
FVC (%predicted)	−0.07	0.68	0.02	0.89	0.07	0.67
FEV_1_ (%predicted)	−0.15	0.37	−0.16	0.36	−0.07	0.66
FEV_1_/FVC	−0.20	0.23	−0.18	0.28	−0.13	0.42
FEF_25−75%_ (%predicted)	−0.21	0.22	−0.25	0.13	−0.25	0.13
PEF (%predicted)	−0.19	0.25	−0.20	0.23	−0.12	0.49

*A p-value < 0.05 indicates statistical significance*

(^*^p = 0.05–0.1,

** *p < 0.05). WC/Ht, waist circumference-to-height ratio; r, correlation coefficient; 6-MWT distance, 6-minute walk test distance; SD, standard deviation; MIP, maximal inspiratory pressure; MEP, maximal expiratory pressure; FVC, forced vital capacity; FEV1, forced expiratory volume in 1 s; FEF_25−75%_, forced expiratory flow rate within 25–75% of vital capacity; PEF, peak expiratory flow. The bold italic means a p-value < 0.1*.

The pulmonary function variables that correlated with obesity indices with a *p*-value <0.1 in univariate analysis were included in stepwise multiple linear regression analysis. That analysis revealed WC/Ht to be the only independent variable influencing the 6-MWT distance with an adjusted *R*^2^ of 0.24, *B* (unstandardized coefficients) of −573 [95% confidence interval (CI): −902.75 to −243.96], and a *p*-value of 0.001. By way of translation, this means that an increase in WC/Ht by 0.1 reduced the 6-minute walking distance by 57.3 m. BMI was the only independent variable that affected the %predicted MEP with an adjusted *R*^2^, *B*, and *p*-value of 0.08, −1.25 (95% CI: −2.46 to −0.49), and 0.04, respectively.

## Discussion

Studies comparing the 6-MWT distance between children and adolescents with obesity and normal-weight age- and gender-matched subjects are scarce, but the results of those few studies and those of the present study are relatively similar. Our study showed that the obesity group had a significantly shorter 6-MWT distance compared to the normal-weight group, which is consistent with previous studies (6, 8, 9).

The 6-MWT is a safe, simple, inexpensive, well-tolerated, and standardized test that has good reproducibility and validity in obese children and adolescents (8). It also reflects a functional exercise level for performing activities of daily living (4, 24). Therefore, the 6-MWT should be recommended for evaluating physical capacity in obese children and adolescents who usually have shortness of breath during physical activities.

The explanation for the observed shorter 6-MWT distance in children and adolescents with obesity is likely to be the excessive fat deposition that causes reduced chest wall compliance and impaired lung mechanics that collectively lead to decreased functional capacity (9, 30). In addition, these individuals have more body mass to move, which requires more energy and mechanical costs (31–33).

Studies in obesity indices that influence functional fitness specific to 6-MWT distance in obese children and adolescents are limited, and the WC/Ht ratio, which is an abdominal obesity index, is rarely included as a variable. To our knowledge, only the recent study by Axley et al. (10) used WC/Ht in their study, and their results showed WC/Ht to be the variable most strongly correlated with 6-MWT distance. Consistent with those findings, our study found WC/Ht to be the only independent predictor of altered 6-MWT distance in multiple linear regression analysis even though BMI, BMI *z*-score, CC, WC, and WC/Ht were all found to be significantly associated with 6-MWT distance in univariate analysis.

Several mechanisms of impaired lung function by abdominal obesity have been proposed (12). The important mechanism is physiological effect of excessive fat deposition in the diaphragm and abdominal visceral organs, which causes a mechanical effect on diaphragmatic movement (34). Another possible reason is systemic inflammation by visceral adipose tissue-released cytokines (35, 36).

The results of the present study and the study by Axley et al. (10) indicate that the WC/Ht ratio exerts significant effect on functional capacity compared to WC alone in obese children and adolescents. The reason might be that WC is strongly linked with age and gender in children and adolescents (37). Increased WC in children and adolescents who are growing is not only from abdominal fat but also from growth effect of increasing age. However, in adults, increased WC reflects more abdominal fat deposition. The WC/Ht ratio is also better than both WC and BMI for indicating adiposity in children and adolescents (38). More studies are required to support the strong association between WC/Ht and functional capacity in these patients. In the future, we intend to determine the cutoff value of WC/Ht and use it as a tool to predict low functional capacity in obese children and adolescents.

Our study found the FEV_1_/FVC, FEF_25−75%_, and PEF values in the obese group to be significantly lower than those in the normal-weight group, but there was no significant difference in FVC, FEV_1_, or RMS between groups. BMI was the only independent variable that affected %predicted MEP, but the correlation was of low level.

The results of RMS between the obese and normal-weight groups in children and adolescents and even in adults (39) remain controversial. The results of our study correspond with those reported by Charususin et al. (40) and Costa et al. (41), but there was a study that reported the MIP in the normal-weight group to be significantly higher (42). There are few studies of the RMS in children and adolescents with obesity, so further study is needed.

Our study supports most previous studies (43, 44) that reported decreased FEV_1_/FVC without airway obstruction problem in obese children and adolescents. This can be explained by the airway dysanapsis theory (43–45), which is defined as the disproportionate overgrowth of lung tissue compared with airway size. Regarding FVC and FEV_1_, recent meta-analysis and systematic reviews (43, 44) reported no difference between the obese and normal-weight groups, which is consistent with the results of our study. A systematic review that was conducted in 2020 (44) found that 47% of studies in obese children and adolescents reported a lower FEF_25−75%_ or a negative association with obesity, but 44% found no difference between or association with obesity.

### Limitations

This study has some mentionable limitations. First, the size of our study population was relatively small, which means that a comparison between genders relative to the association between obesity indices and PFTs (44) could not be performed. Second, body fat was not measured by direct methods, such as bioelectrical impedance analysis (BIA) or dual-energy X-ray absorptiometry (DEXA) because we wanted to use simple and practical methods of measuring adiposity that can be easily and affordably employed in routine clinical practice.

## Conclusion

Children and adolescents with obesity had a significantly shorter 6-MWT distance than children and adolescents in the normal-weight group. The 6-MWT reflects functional capacity to perform daily physical activities. Our results suggest the use of the 6-MWT for evaluation and monitoring of functional capacity in obese children and adolescents, which is a group that routinely experiences shortness of breath during physical activities. In addition, WC/Ht was shown to be the best predictor of abdominal obesity indices that influenced functional fitness in obese children and adolescents. Therefore, WC/Ht may be used as a tool to identify low functional capacity in these patients in both research and clinical settings.

## Data Availability Statement

The original contributions presented in the study are included in the article/supplementary material, further inquiries can be directed to the corresponding author/s.

## Ethics Statement

The studies involving human participants were reviewed and approved by Institutional Review Board of Siriraj Hospital (Si 848/2020). Written informed consent to participate in this study was provided by the participants' legal guardian/next of kin.

## Author Contributions

KU contributed to study conception, study design, statistical analysis, and manuscript preparation. TT recruited study participants, conducted fieldwork, and performed data collection. AN and PT contributed to study conception and design. PC and KM conducted fieldwork and performed data collection. All authors contributed to the article and approved the submitted version.

## Funding

This research project was supported by a grant from the Siriraj Routine to Research Management Fund of the Faculty of Medicine Siriraj Hospital, Mahidol University, Bangkok, Thailand (Grant No. R2R541/20).

## Conflict of Interest

The authors declare that the research was conducted in the absence of any commercial or financial relationships that could be construed as a potential conflict of interest.

## Publisher's Note

All claims expressed in this article are solely those of the authors and do not necessarily represent those of their affiliated organizations, or those of the publisher, the editors and the reviewers. Any product that may be evaluated in this article, or claim that may be made by its manufacturer, is not guaranteed or endorsed by the publisher.
